# Inferring Drosophila gap gene regulatory network: pattern analysis of simulated gene expression profiles and stability analysis

**DOI:** 10.1186/1756-0500-2-256

**Published:** 2009-12-16

**Authors:** Yves Fomekong-Nanfack, Marten Postma, Jaap A Kaandorp

**Affiliations:** 1 Section Computational Science, Faculty of Science University of Amsterdam. Science Park 107, 1078 XJ, Amsterdam, The Netherlands

## Abstract

**Background:**

Inference of gene regulatory networks (GRNs) requires accurate data, a method to simulate the expression patterns and an efficient optimization algorithm to estimate the unknown parameters. Using this approach it is possible to obtain alternative circuits without making any *a priori *assumptions about the interactions, which all simulate the observed patterns. It is important to analyze the properties of the circuits.

**Findings:**

We have analyzed the simulated gene expression patterns of previously obtained circuits that describe gap gene dynamics during early *Drosophila melanogaster *embryogenesis. Using hierarchical clustering we show that amplitude variation and defects observed in the simulated gene expression patterns are linked to similar circuits, which can be grouped. Furthermore, analysis of the long-term dynamics revealed four main dynamical attractors comprising stable patterns and oscillatory patterns. In addition, we also performed a correlation analysis on the parameters showing an intricate correlation pattern.

**Conclusions:**

The analysis demonstrates that the obtained gap gene circuits are not unique showing variable long-term dynamics and highly correlating scattered parameters. Furthermore, although the model can simulate the pattern up to gastrulation and confirms several of the known regulatory interactions, it does not reproduce the transient expression of all gap genes as observed experimentally. We suggest that the shortcomings of the model may be caused by overfitting, incomplete model description and/or missing data.

## Introduction

A biological system that has been extensively studied is the segmentation mechanism of early development in *Drosophila melanogaster *(see [1] for review). At early stage, a cascade of maternal and zygotic genes is activated in the syncytial embryo that subdivides the ectoderm into smaller domains. First, maternal morphogenes such as *bicoid (bcd)*, caudal (cad) and *hunchback (hb) *activate zygotic gap genes such as *hb*, *giant *(*gt*), *Krüppel *(*Kr*), *knirps *(*kni*), or *tailles *(*tll*), which in turn will activate the pair rule genes. The pair rule genes will regulate segment polarity genes and Hox genes, which both control the differentiation of each segment of the future embryo [1].

The gap gene circuit has been extensively investigated using mathematical models [2,3]. In all cases, the goal was to derive the regulatory interactions that control gene expression. The gene circuit approach [4] combined with a parameter optimization method allowed to infer gene regulatory interactions directly from experimental spatio-temporal gene expression data [5,6]. In all cases the optimization involved minimization of the difference between observed data and simulated data. Previous studies [4,7,8] have analyzed the obtained gene circuits essentially by visual inspection of the simulated patterns, mainly because of an insufficient number of circuits. Fomekong et al. [8] proposed a faster optimization method that yielded a higher number of circuits, allowing for a more detailed analysis. Finding a set of parameters that reproduces the observed data does not necessary imply that the network structure has been identified correctly, or that the underlying pattern formation mechanism of the system has been revealed [9,10]. For some systems, the network structure itself inherently leads to robust pattern formation and is weakly depended on the specific parameter values [11,12]. Inference may lead to a unique network, however for many cases many circuits with different topologies and scattered parameter values are found. It is necessary to further analyze these circuits and discriminate between realistic and non-realistic circuits based on other criteria [13,14].

We have analyzed the simulated patterns and parameters of the circuits that were obtained previously using descriptive statistics and stability analysis [8,15]. The incompleteness of the available experimental data, the complexity and the non-linearity of the model and the large number of unknown parameters potentially leading to over-fitting makes the reverse engineering problem challenging. It might lead to circuits with different regulatory interactions or variability in the simulated patterns and dynamical behavior.

## Findings

### Simulated profiles

**Figure 1:**
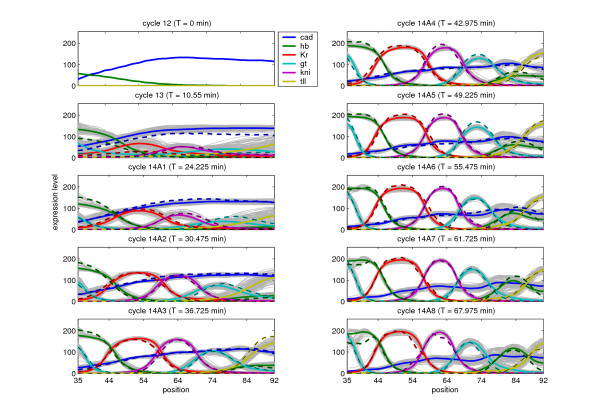
**Expression profiles of the 101 gap gene circuits at different time points**. Individual gene profiles are shown in light gray and the average profile of that gene at a specific time point is plotted using a colored solid lines. The x-axis corresponds to 35-92% of the A-P position and the y-axis describes the expression level influorescence units. Each panel corresponds to one of the 10 time points (12, 13 and 14A1-14A8) for which data are available. The experimentally measured expression profiles are plotted using colored dashed lines.

Although all circuits show relatively good fits with respect to the data (see Figure 1), small features, like bumps, dips and other variations in the expression profiles at gastrulation time are observed (see Additional file 1). These features are not observed in the data, and may represent circuits that are not biologically realistic. We performed a hierarchical cluster analysis on the profiles to identify groups that share deviant features. By statistical comparison of the parameters among the different groups using a T-test we find parameters that may explain the observed features.

**Figure 2:**
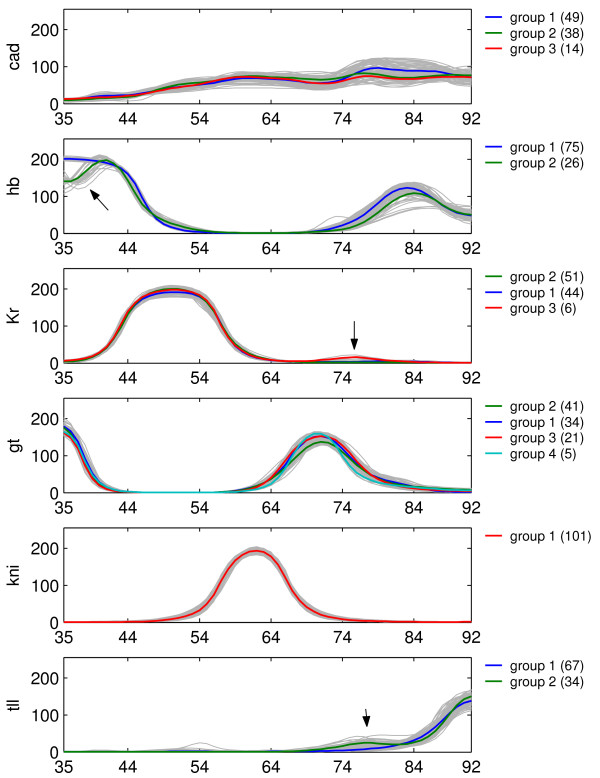
**Hierarchical clustering of simulated profiles at T = 68.1 min**. The mean expression profile of the groups obtained from clustering are shown using colored solid lines. The individual expression profiles of each circuit are shown in gray.

Figure 2 shows the clustered simulated profiles where we observe four main pattern groups described as follows: group 1: no defection. group 2: *hb *showing a dip in the anterior domain. group 3: *tll *showing a shoulder. group 4: *Kr *showing an extra bump.

**Figure 3:**
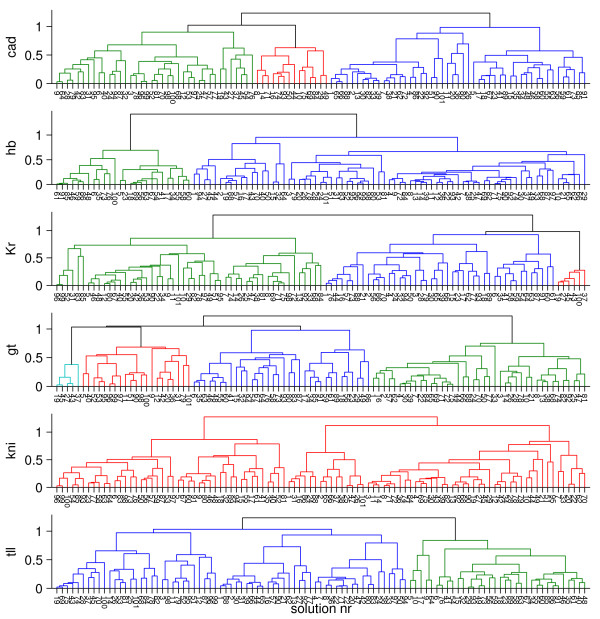
**Dendrograms obtained from hierarchical clustering of the simulated profiles at T = 68.1 min**. Each Individual tree diagram corresponds to the distribution of all the profiles obtained from the different circuits for a single gene. In each tree, the circuits belonging to the same cluster are grouped.

We noticed that some of the clusters share the same circuits as shown in Figure 3. We observe that the group with the *hb*-anterior dip largely overlaps with the *tll *bump cluster and also with one of the *gt *clusters. This means that the features in *hb*, *tll and gt *share a common circuit topology (see Venn-diagram Figure S1 in Additional file 1).

The differences between the profiles may be explained by variability in circuit topology or by differences in parameter magnitude. By comparing the parameters of the different groups using a T-test, we find that parameters of group 2 and group 3 do not show any significant differences, and therefore are combined into one group 2-3. Comparison of group 1 and group 2-3 yields three parameters:  (Table 1). In Group 2-3 Gt represses *hb *and causes the anterior *hb *dip. Also, *tll *activation by Gt is considerably decreased, leading to higher production of *tll*, which causes the *tll*-bump. Comparison of group 1 and 4 shows that *Kr *autoactivation is strong and repression by Gt is weak within group 4 (Table 2). This combination causes a local increased production of *Kr *on the domain where Gt is expressed. A consequence of the strong autoactivation would be a higher level of *Kr *all along the A-P axis. This is prevented by increased repression through Hb and Kni. Also, the weaker production rate of *Kr *compensates for the strong autoactivation.

**Table 1:** Parameter differences between circuits of group 1 and circuits of group 2-3.

parameter differences between circuits of group 1 and group 2-3				
***θ***	***m*1**	***m*2**	***dm***	***p *- *value***

*tll *→ *cad*	-0.0171377	-0.02491	-0.00777229	1.04553*e *- 010
*hb *→ *hb*	0.0217064	0.015097	-0.00660943	1.34364*e *- 011
*gt *→ *hb*	0.0122061	-0.00414659	-0.0163527	0
*kni *→ *hb*	-0.128935	-0.0747608	0.0541739	5.28773*e *- 009
*Kr *→ *Kr*	0.0161343	0.0227917	0.00665739	4.79087*e *- 007
*gt *→ *Kr*	-0.0528814	-0.027446	0.0254355	2.71076*e *- 010
*hb *→ *gt*	-0.003434	0.00397726	0.00741126	8.47311*e *- 011
*gt *→ *gt*	0.0132754	0.0164244	0.00314898	2.4076*e *- 006
*tll *→ *gt*	-0.0155584	-0.0438868	-0.0283284	1.11022*e *- 015
*gt *→ *tll*	-0.0252096	-0.00316618	0.0220435	3.74904*e *- 007
*m *→ *Kr*	0.0525071	0.0306038	-0.0219033	6.33614*e *- 007
*m *→ *gt*	0.0703723	0.0261228	-0.0442495	8.70947*e *- 009

*T*-test comparison of circuit parameters belonging to a group with a normal pattern without any defection (group 1) and a solution for which *hb *has a dip and *tll *a bump (group 2 and 3).

In groups 2 and 3, Gt represses *hb*, causing the dip observed at anterior *hb*. Also, Hb activates *gt *(contrarily to group 1). Consequently, there should be an increased production of anterior *gt *and something should locally repress *gt *to keep it at its normal level. At this position, Tll is the gene that controls *gt *expression level, and one way to keep it constant would be to increase the repression weight.

**Table 2:** Parameters' differences between circuits of group 1 and circuits of group 4.

parameter differences between circuits of group 1 and group 4				
***θ***	***m*1**	***m*2**	***dm***	***p *- *value***

*hb *→ *Kr*	-0.00322924	-0.022085	-0.0188558	2.06398*e *- 008
*Kr *→ *Kr*	0.0161343	0.0495102	0.0333758	2.22045*e *- 016
*gt *→ *Kr*	-0.0528814	-0.000873188	0.0520082	1.91889*e *- 005
*kni *→ *Kr*	-0.0100416	-0.0509584	-0.0409168	6.4837*e *- 014
*gt *→ *gt*	0.0132754	0.0213547	0.00807934	1.41633*e *- 005
*kni *→ *gt*	0.00206418	-0.00166141	-0.00372559	0.000687105
*R_Kr_*	21.2186	14.312	-6.90662	0.000229012

*T*-test comparison of solution parameters belonging to a group with a normal pattern without any defection (group 1) and a solution for which Kr shows a posterior bump (group 4).

### Pattern stability at later times

During gastrulation most of the gap domains, maternal *bcd *and *cad *disappear within 30 min. The anterior *hb *domain disappears rapidly during gastrulation [16], while posterior *hb *domain can still be detected for a few more hours until the end of germ band extension [17]. Central *Kr *domain decays rapidly after the onset of gastrulation [18,19]. Posterior *gt *domain disappears rapidly during gastrulation while the anterior domains persist for a few hours but change quite drastically and become involved in organ formation [20-22]. The entire *kni *domain and the posterior domain of *tll *disappear rapidly after gastrulation [23,24].

**Figure 4:**
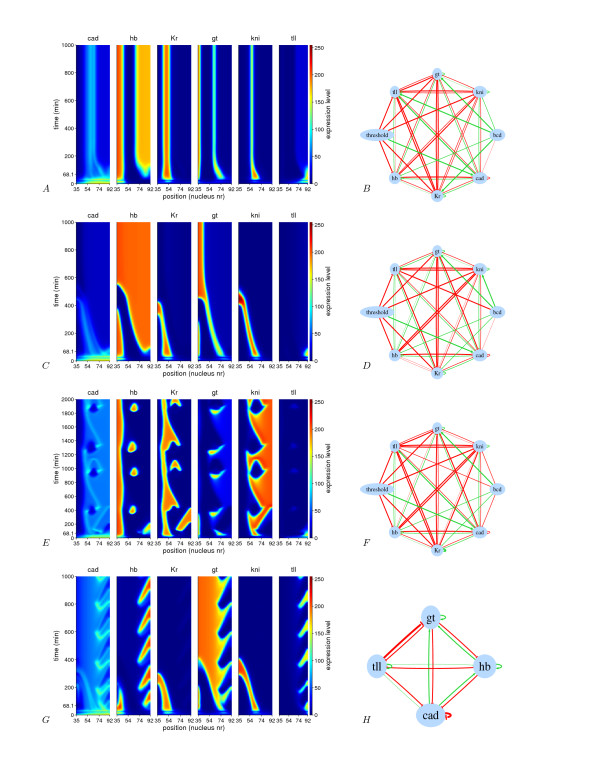
**Spatio-temporal surface plots showing the behavior of four different circuits at later times, and on the right the corresponding circuits**. Surface plots (panel a, c, e and g) represent the main types of patterns observed: a) Stable pattern with reminiscent pattern (Group I). c) stable pattern with a large hb suppressing all genes except gt (Group II). e) Oscillatory pattern where all genes except Tll oscillate (Group III). g) An oscillatory pattern where all genes except Kr and kni oscillate at the posterior (Group IV). In panel h the reduced circuit is shown. Only the connections that correlate with this particular pattern are shown. In this circuit typical oscillatory motifs can be recognized. Edges between two vertices indicate activation (green) or repression (red). The edge thickness is proportional to the absolute weight of the interaction.

The long-term dynamics of the circuits should show if the model is able to predict the disappearance of the gap gene domains, and provide information about the asymptotic stability of the model and potentially gives its attractors. The parameters were obtained by fitting the model to real data until gastrulation time. To study long term dynamics we simulated all circuits for an extended period (see Methods, Additional file 1 and Additional files 2, 3, 4, 5 where long term dynamic movies are shown). We classified the behavior into the following groups:

1. stable patterns: 64 circuits, where *tll *and *cad *domains disappear completely in most cases. This group is composed of three sub-groups

(a) 9 circuits show a rudimentary gap gene pattern with all gene domains more or less well defined. (Figures 4-A, B).

(b) 27 circuits develop an uniform *hb *domain that covers the whole embryo. (Figures 4-C, D).

(c) 28 circuits show variable stable patterns with expanding or disappearing domains.

2. Oscillatory patterns: (37 circuits) with the two sub-groups:

(a) 18 circuits with *cad*, *hb*, *gt *and *tll *showing posterior oscillations, while *Kr *and *kni *domains disappear. (Figures 4-E, F).

(b) 19 circuits where all genes oscillate but *tll*, which disappears (Figures 4-G, H).

**Figure 5:**
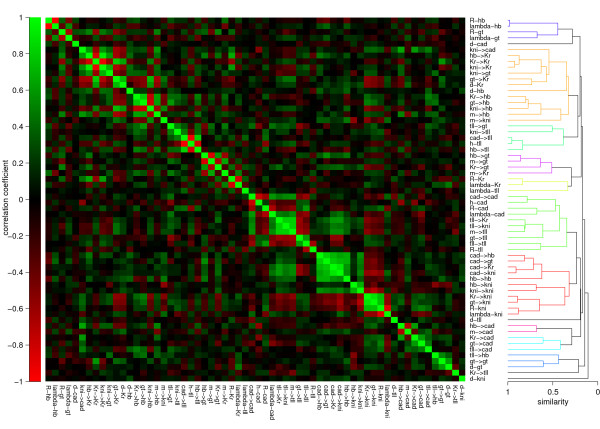
**Parameter correlation matrix**. Left: Matrix showing the pairwise correlation; the colour scale goes from intensive red (strong negative correlation) to bright green (positive correlation). The correlation matrix shows that there exist many pair wise correlations that tend to form clusters. Right: The absolute value of the correlation coefficients are used as a similarity measure to cluster the parameters, which is presented as a dendrogram. The parameters are sorted according to the dendrogram.

We have compared parameters using T-tests and the average circuit topology (see Table 3, 4 and 5). From Table 3, we see that the main difference between the two stable patterns is the strong *hb *autoactivation in the group with expanded *hb*. In some of the oscillatory circuits (Figure 4-H), we observed a basic motif composed of autoactivation and negative feedback loops. It can be shown theoretically that the minimal requirement for oscillations to occur in a two-gene network is that an activator activates its repressor and also itself. Nevertheless, the positive and negative feedback loops may be indirect and also the actual parameter values may prevent the formation of oscillations even if the minimal requirement for oscillations is present. In the first oscillatory group we observe the basic motif for oscillation between Hb and Gt (Figure 5-H). In the data we observe that the anterior *hb *peak slightly collapses, however it collapses more at the position of the anterior *gt *peak. In a number of circuits the fit to Hb is improved by repression of *hb *by Gt (group 2 cluster analysis). Almost all members of this group show oscillations. Hb in this group has an intermediate autoactivation (the group with strong autoactivation does not show oscillations) and Cad activates *hb *leading to constitutive activation of posterior *hb*. Next to the *hb*-gt oscillatory motif we see similar motifs with Tll. When negative feedback interactions are removed we observe that the corresponding gene does not oscillate any longer. Although the connections in these motifs are weak the behavior at later times is strongly affected.

**Table 3:** Comparison of an average network with stable pattern formation(group I) against a network with a stable pattern and with expanded Hb domain (group II).

Network Differences				
***θ***	***m*1**	***m*2**	***dm***	***t***

*hb *→ *hb*	0.023993	0.020258	-0.00373504	0.0016962
*bcd *→ *kni*	0.0448947	-0.0123351	-0.0572298	2.85189*e *- 006

The table summarizes the list of parameters that are significantly different (mean *mi*, difference between mean *dm *and their p-value from the T-test *t*. The parameter difference found between Group I and II are the strength of hb autoactivation and the activation/repression of kni by Bcd. [see Tab. S1 in Additional file 1, where networks diagrams are shown.]

**Table 4:** Comparison of an average network with a stable pattern group (group II) against oscillatory pattern (group III).

Network Differences				
***θ***	***m*1**	***m*2**	***dm***	***t***

*hb *→ *hb*	0.0202833	0.023993	0.00370971	0.000132337
*kni *→ *hb*	-0.148545	-0.0960403	0.0525049	0.000899982
*hb *→ *gt*	-0.00730634	0.000129385	0.00743572	0.00103439
*bcd *→ *kni*	-0.000129675	0.0448947	0.0450244	0.000589936

The table summarizes the list of parameters that are significantly different. Group II is stabilized by the over production of *hb *(activated by Gt). [see Tab. S2 in Additional file 1, where networks diagrams are shown.]

**Table 5:** Comparison of an average network of the two groups with oscillatory pattern (group III vs. group IV). [Supplementary-material S1]

Network Differences				
***θ***	***m*1**	***m*2**	***dm***	***t***

*hb *→ *cad*	-0.0479759	-0.0239867	0.0239891	0.000701299
*Tll *→ *cad*	-0.0197665	-0.0261618	-0.00639534	0.00334701
*hb *→ *hb*	0.0202833	0.0133955	-0.00688781	1.11532*e *- 005
*gt *→ *hb*	0.0131477	-0.00553095	-0.0186786	4.33042*e *- 011
*kni *→ *hb*	-0.148545	-0.0728052	0.0757399	7.19654*e *- 005
*hb *→ *gt*	-0.00730634	0.00505889	0.0123652	3.60571*e *- 006
*Kr *→ *gt*	-0.103984	-0.0585162	0.0454676	0.000300942
*Tll *→ *gt*	-0.0107778	-0.0464788	-0.035701	6.88338*e *- 014
*gt *→ *Tll*	-0.036005	-0.00193247	0.0340725	0.000156841
*bcd *→ *cad*	-0.014402	-0.0389897	-0.0245877	7.47514*e *- 005
*bcd *→ *Kr*	0.0576209	0.0287058	-0.0289151	0.000306426
*bcd *→ *gt*	0.0957429	0.0223168	-0.0734261	6.08573*e *- 005
*bcd *→ *kni*	-0.000129675	0.0630306	0.0631603	0.000139936

The table summarizes the list of parameters that are significantly different. All the parameters in the two groups have the predict the same regulatory interactions (but, with some extent,  [see Tab. S3 in Additional file S11, where networks diagrams are shown.]

### Parameter correlations

From the two previous analyses and T-tests, we see that parameters differ from circuit to circuit leading to different behaviour. This might be a sign of overfitting, which can be determined by looking at the correlation matrix of all the parameters (Figure 5). Because of compensation mechanisms parameters may not be identifiable. Examples of these are promoter and decay rates, which both scale the expression profile.

Furthermore, the input weights on a single gene can also compensate each other. If a positive input on a gene becomes stronger, increasing negative weights or decreasing positive weights can adjust for the increased total input, such that the total input on that gene is not altered much. However, these correlation patterns may be more intricate [see Additional file 1 for an extended correlation analysis].

## Discussion

It seems difficult to determine which of the circuits have the "correct" topology. From the clustering of the gastrulation profiles, we could have considered that only circuits without defects should be taken into account, but we see that it is not that trivial since the difference from circuit to circuit is not only based on the regulatory interaction type, but also their strength. None of the circuits predicted the disappearance of the gap genes during gastrulation, this may be related to missing mechanisms like degradation of maternal genes. The long-term dynamics of the circuits show that the patterns converge to four main attractors. This difference in convergence may be explained by differences in a few, but also the presence of certain motifs. More intriguing, one of the attractors resembles the gastrulation pattern and circuits falling into this group have interactions more consistent with experimental evidence. Combined with the well defined parameters obtained from the correlation analysis [12], the following gap gene interactions consistent with literature were derived:

1. All the gap gene are activated by Cad.

2. All the gap gene but kni are activated by Bcd.

3. Hb, Kr gene have an auto-activation.

4. kni does not have a auto-repression, but certitude on auto-activation can not be deduced (strong correlation coefficient with most parameters)

5. Mutual repression between Hb and Kni

6. Mutual repression between Gt and Kr

These interactions are consistent with the regulatory mechanism proposed in [4] as well as those obtained in early literature [19-21, 25-29] and previous analysis [10]. Out of all the circuits, only 4 have a very good patterns and the mentioned regulatory interactions [circuits 20, 31, 82 and 101, see Additional file 6]. The alternative interactions proposed by the other circuits are a consequence of overfitting, incomplete data and incomplete model structure.

For example biological evidence suggests that the anterior *hb *dip is caused by different early and late regulation mechanisms, which is not included in the current model, consequently the optimization predicts for many circuits suppression of *hb *by Gt to mimic this data feature. Furthermore the model tries to reproduce the experimentally observed decrease of *cad *by introducing negative feedback through the gap genes.

Jaeger et al. [4] suggested that the anterior shift of posterior domains after cycle 14A is caused by asymmetric repression of the gap genes. All the current circuits reproduce the shift, but from the current analysis, it seems that the shift is not necessary a consequence of the asymmetric repression triggered by Hb. In many circuits we see that the shift of these domains continues to progress and leads to domain expansion or disappearance of other domains. The shift seems to correlate strongly with the posterior hb domain. The posterior *hb *domain develops later than the other domains, and represses *gt*, *kni *and *Kr*. In the circuits where the posterior *hb *domain continues to expand and in the end forms an almost uniform domain at steady state that covers the whole embryo; the anterior gt domain remains and *Kr*, *kni*, *tll *and *cad *all disappear. This phenomenon is caused by strong hb autoactivation, the other gap genes are not able to balance *hb *expression. The anterior *gt *domain remains because of maternal activation by Bcd and weak repression by Hb.

Schroder et al. [30] suggested that autoactivation is involved in maintenance of gap gene expression and sharpening of gap domain boundaries [15]. Although this might be true, strong autoactivation also affects pattern stability later on during gastrulation, making it more difficult for domains to fade. The inability of the circuits to predict transient expression suggests that either an additional mechanism is missing in the model or that the optimization failed to capture the dynamics.

## Competing interests

The authors declare that they have no competing interests.

## Authors' contributions

All authors participated in the design of the study, and wrote the manuscript together. YF-N and MP conducted the analysis of data and simulations, and made the figures. All authors read and approved the final manuscript.

## Supplementary Material

Additional file 1 **Additional statistics**. This file (GapGenePatternAnalysis BMCRN AddFile1) contains the material, which is not given in the paper due to the space limitations. In Section 1, models and methods are described. Section 2 gives a complete description of the simulated profiles. Section 3 complete the long term dynamics comparison with additional table-figures and Section 4 presents a complete correlation analysis. Click here for file

Additional file 2 **Stable pattern with reminiscent pattern**. Movies displaying the long term behaviour of a circuit showing a stable pattern with reminiscent gap gene pattern. Click here for file

Additional file 3 **Stable pattern with a large hb suppressing all genes except gt continuously expanding to the left**. Movies displaying the long term behaviour of a circuit showing a stable pattern with a large hb suppressing all genes except gt continuously expanding to the left. Click here for file

Additional file 4 **Oscillatory pattern where all genes except Tll oscillate**. Movies displaying the long term behaviour of a circuit showing an oscillatory pattern where all genes except Tll oscillate. Click here for file

Additional file 5 **Oscillatory pattern where all genes except Kr and kni oscillate at the posterior**. Movies displaying the long term behaviour of a circuit showing an oscillatory pattern where all genes except Kr and kni oscillate at the posterior. Click here for file

Additional file 6 **Details parameters set of the 101 circuits**. The data provided in the table contains a complete list of all the 101 circuits with their corresponding parameters. Click here for file
