# Supplementary material for: Inferring Drosophila gap gene regulatory network: pattern analysis of simulated gene expression profiles and stability analysis
Source: BMC Res Notes. 2009 Dec 16;2:256. doi: 10.1186/1756-0500-2-256 (PMC2808311; doi:10.1186/1756-0500-2-256)
Supplement: Additional file 1 — Additional statistics. This file (GapGenePatternAnalysis BMCRN AddFile1) contains the material, which is not given in the paper due to the space limitations. In Section 1, models and methods are described. Section 2 gives a complete description of the simulated profiles. Section 3 complete the long term dynamics comparison with additional table-figures and Section 4 presents a complete correlation analysis. [file 1756-0500-2-256-S1.PDF]

Supplementary material for the paper

# Inferring *Drosophila* gap gene regulatory network: pattern analysis of simulated gene expression profiles and stability analysis

Yves Fomekong-Nanfack [1], Marten Postma[1], Jaap Kaandorp [1,a]
[1]Section Computational Science, Faculty of Science University of Amsterdam.
Science Park 107 ,1078 XJ Amsterdam The Netherlands.
[a]corresponding author E-mail: J.A.Kaandorp@uva.nl

# 1 Methods

## 1.1 Inference of the Gap Gene model

The gap gene circuits analyzed in this paper were presented by Jaeger et al. [1] and Fomekong-Nanfack et al. [2]. In both cases, the inference was performed using the same quantitative data, the same model but different parameter estimation methods.

**Quantitative data** used are available online in the FlyEx database http://urchin.spbcas.ru/flyex or http://flyex.ams.sunysb.edu/flyex. The database presents a collection of quantitative data obtained from fluorescently stained wild-type embryos for Eve protein and two other genes [3]. Data were obtained by applying different image processing strategies [4–6]. The embryos are for different time ranging from cycle 7 to cycle 14A. In the simulation, data obtained at cycle 12 were used as initial conditions. For the genes *Kr, gt, kni, Tll* these are very close to zero and set to 0 in the simulations.

**Mathematical model** of gap gene considers the 35% to 92% of the A-P axis of an embryo. It is reduced to a one-dimensional discrete model where nuclei are aligned horizontally. The model focuses on the development between cycle 13 and cycle 14A8, before gastrulation (71.1 min). Three rules describe the mechanism during that phase: interphase, mitosis and division [7]. Interphase and mitosis are continuous stages describing the dynamic of protein variation of a gene within a nucleus. The division is a discrete process describing the division of a nucleus in two. Mitosis, arising before division, differs from interphase by the absence of protein synthesis. The resulting model is a system of 180 equations before division and 348 equations after, with a total of 66 unknown parameters written as:

$$\frac{dg_i^a(t)}{dt} = \begin{cases} R_a \Phi_a \left( \sum_{b=1}^{N_g} W_a^b g_i^b + m_a g_i^{bcd} + h_a \right) \\ -\lambda_a g_i^a \\ +D_a \left( g_{i+1}^a - 2g_i^a + g_{i-1}^a \right) \end{cases} \tag{1}$$

where $N_g$ denotes the number of genes or gene products involved and $\Phi$ is a sigmoid function with range (0,1). $g_i^a(t)$ represents the concentration level at time $t$ of gene $a$ in nucleus $i$ with $1 \le i \le N$ and $N$ the number of nuclei during a cleavage cycle. The concentration, $g_i^{bcd}$, of the maternal gene *bicoid* is taken from experimental observations and is kept constant in time during the simulation. The parameters are: the regulatory weight matrix $W_a^b$, describing the influence of gene $b$ on gene $a$, the production rate $R_a$, the activation threshold $h_a$ for $\Phi$, the decay rate $\lambda_a$, the diffusion coefficient

$D_a$, and the regulatory influence $bcd_a$.

**Parameter estimation** was performed by two different strategies. Jaeger et al. [1] have used a parallel simulated annealing (PLSA) algorithm as described in [8], originally proposed by Lam [9,10]. The expensive computational time required by PLSA could only lead to 10 gap gene circuits with good solution's quality and patterns behavior. Later on, Fomekong-Nanfack et al. [2] proposed 101 gap gene circuits obtained using hybrid methods composed of an stochastic ranking evolution strategy [11,12] followed by direct search [13–15]. The large number of solution could be obtained because of the reasonably low computational time of their method (8h on single processor) compare to PLSA (1 to 5 days on 10 parallel CPU), but leading to the same quality of solution. In both cases, the chosen cost-function is the least-squares of the difference of the simulated and the observed data:

$$E(\theta) = \sum_{i,t} (g_i^a(t,\theta)_{model} - g_i^a(t)_{data})^2, \tag{2}$$

with $\theta$ the parameter vector to which a constraint or penalty function is added. An explicit search-space constraint is given for parameters $R_a$, $\lambda_a$ and $D_a$. For the parameters $W_a^b$, $bcd_a$ and $h_a$ a collective penalty function is used ( [16]) to restrict the function value of $\Phi$ to the domain $[\Lambda, 1-\Lambda]$ with $\Lambda$ a small parameter (in this study taken to be 0.001). The root mean square (RMS) described by Reinitz et al. is used ( [16]) as a measure of the quality of a model solution for a given set of parameters:

$$\text{RMS} = \sqrt{\frac{E(\theta)}{N_d}} \tag{3}$$

where $E(\theta)$ is given by Equation ( 2) and $N_d$ is the number of data points.

### 1.2 Statistical analysis

A correlation matrix shows the degree of association between two parameters. The parameter values are centered on the mean and computed using the Pearson correlation.

Clustering algorithms are often used as one of the first gene expression analysis [17]. In the current context, clustering is applied to simulated gene expression obtained from the inferred circuits. The goal is to subdivide the profiles at gastrulation for all the simulated genes in groups, such that dissimilar profiles fall in different clusters. For each gene, 101 profiles at gastrulation time are available. A cluster analysis will highlight all circuits' profile that has a similar pattern. The clustering used here is based on agglomerative hierarchical clustering [18]. Prior to linkage, the profiles are median centered and normalized. Then, a dendrogram relating similar circuits in the same tree is hierarchically constructed based on the average linkage and the absolute correlation coefficient. Similarity between circuits of different clusters on the basis of the parameter is obtained by a $t$-test.

### 1.3 Stability

We assume that at gastrulation time, the system reached is steady state corresponding to the end of cycle 14A8 (where starting simulation time is cleavage cycle 13 ad total simulation corresponds to approximately 71.1 minutes real time development). We therefore simulate the model up to 1000 or 2000 minutes and classified the resulting spatio-temporal patterns qualitatively in terms of the different observed behavior (stable close or not to attractor and oscillation). Using a $t$-test, we compare the parameters between the different groups to find parameters that are significantly different.

## 2 Description of the simulated profiles

**Caudal** The simulated profiles show a lower expression level than the real data, suggesting that the decay coefficient might be too small. The profiles from time point 14A1 to 14A3 show a good fit contrarily to those from 14A4 to 14A8. Cad expression at later times is rather variable. The data also show that caudal collapses slightly overtime, which is not well represented in the model. Late Cad

profile variation might be caused by missing data at the two last time points ( cleavage cycle 14A7 and 14A8). This gives freedom for the fit and allows for repression of caudal by other genes.

**Tailless** The profiles show an overall good fit beside the fact that there were some early time points missing (cleavage cycle 13 to 14A3). However, in some of the circuits there is a small shoulder present at the posterior hunchback peak and sometimes a very small bump at the Kr peak.

**Anterior hunchback** The simulated profiles are higher than the observation at cleavage cycle 13. From cleavage 14A1 to 14A6 the profiles are well fitted against the real data, especially the boundary. At time 14A7 and 14A8 in some cases, a dip is formed in profile.

**Posterior hunchback** The observed profiles in early times are well fitted, however later on from 14A4/14A8 the model has difficulties to represent the retraction of the posterior hunchback peak.

**Krüppel** The observed profiles are well fitted for all time points with the exception of cleavage cycle 13 for which the expression level is much higher than observations. Very little variations appear on the posterior domain and increase slightly the krüppeldomain at time 14A8.

**Anterior giant** The simulated profile shows an overall good fit without any defection.

**Posterior giant** The simulated profile has an overall good fit, only at later times it has minor difficulties to retract. There is some variability in the posterior giant peak.

**Knirps** The observed profile is very well fitted although minor variations appear in the shape of the peak for some gap gene circuits.

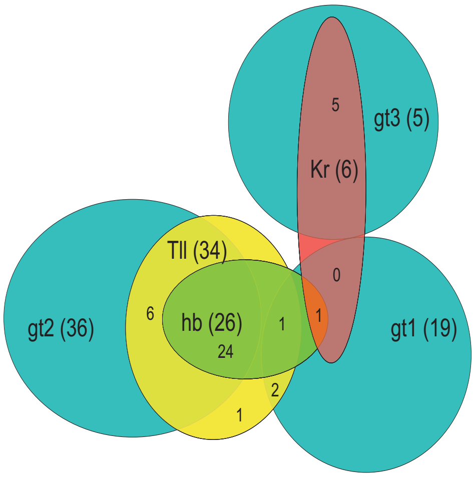


Figure 1: Hypothetically possible logical relations between set of circuits. Each color corresponds to a gene. Only the solutions with a defect are selected. Overlapping sets describe solutions having multiple genes profiles showing that defect.

# 3 Comparision of different groups obtained from the long term dynamics

Network with stable pattern with expanded Hb

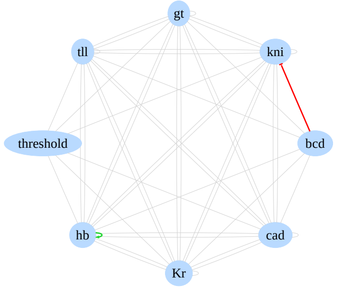


Network with stable pattern

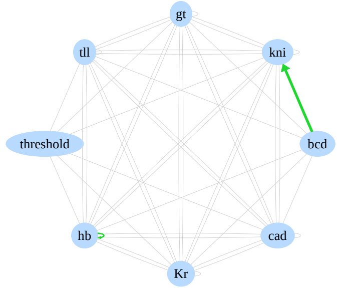


| Network Differences | | | | |
|---|---|---|---|---|
| $\theta$ | $m1$ | $m2$ | $dm$ | $t$ |
| $hb \to hb$ | 0.023993 | 0.020258 | $-0.00373504$ | 0.0016962 |
| $bcd \to kni$ | 0.0448947 | $-0.0123351$ | $-0.0572298$ | $2.85189e-006$ |

Table 1: Comparison of an average network with stable pattern formation(group I) against a network with a stable pattern and with expanded Hb domain (group II). Interactions that are not significantly different between the two groups are shown in light gray. The interactions that are significantly different are shown in colour. The table summarizes the list of parameters that are significantly different (mean $mi$, difference between mean $dm$ and their p-value from the T-test $t$. The parameter difference found between Group I and II are the strength of hb autoactivation and the activation/repression of kni by Bcd.

Network with oscillating pattern of: Cad, Hb, Kr, Gt and Kni

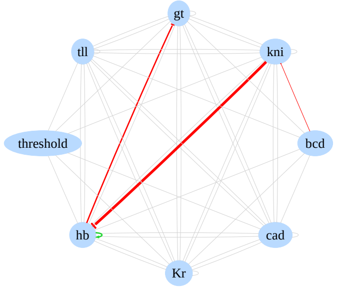


Network with stable pattern with expanded Hb

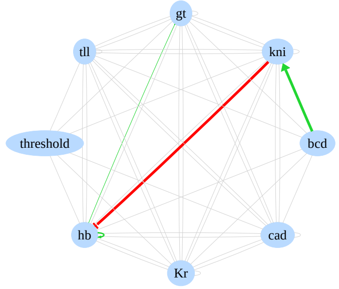


| Network Differences | | | | |
|---|---|---|---|---|
| $\theta$ | $m1$ | $m2$ | $dm$ | $t$ |
| $hb \to hb$ | 0.0202833 | 0.023993 | 0.00370971 | 0.000132337 |
| $kni \to hb$ | $-0.148545$ | $-0.0960403$ | 0.0525049 | 0.000899982 |
| $hb \to gt$ | $-0.00730634$ | 0.000129385 | 0.00743572 | 0.00103439 |
| $bcd \to kni$ | $-0.000129675$ | 0.0448947 | 0.0450244 | 0.000589936 |

Table 2: Comparison of an average network with a stable pattern group (group II) against oscillatory pattern (group III). Interactions that are not significantly different between the two groups are shown in light gray. The interactions that are significantly different are shown in colour. The table summarizes the list of parameters that are significantly different. Group II is stabilized by the over production of $hb$ (activated by Gt).

Network with oscillating pattern of:
Cad, Hb, Kr, Gt and Kni

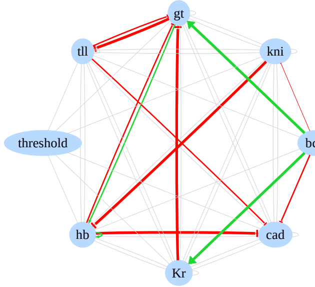


Network with oscillating pattern of:
Cad, Hb, Gt and Tll

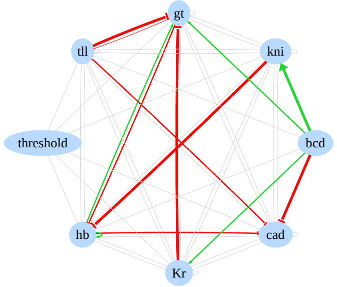


| Network Differences | | | | |
|---|---|---|---|---|
| $\theta$ | $m1$ | $m2$ | $dm$ | $t$ |
| $hb \to cad$ | −0.0479759 | −0.0239867 | 0.0239891 | 0.000701299 |
| $Tll \to cad$ | −0.0197665 | −0.0261618 | −0.00639534 | 0.00334701 |
| $hb \to hb$ | 0.0202833 | 0.0133955 | −0.00688781 | $1.11532e-005$ |
| $gt \to hb$ | 0.0131477 | −0.00553095 | −0.0186786 | $4.33042e-011$ |
| $kni \to hb$ | −0.148545 | −0.0728052 | 0.0757399 | $7.19654e-005$ |
| $hb \to gt$ | −0.00730634 | 0.00505889 | 0.0123652 | $3.60571e-006$ |
| $Kr \to gt$ | −0.103984 | −0.0585162 | 0.0454676 | 0.000300942 |
| $Tll \to gt$ | −0.0107778 | −0.0464788 | −0.035701 | $6.88338e-014$ |
| $gt \to Tll$ | −0.036005 | −0.00193247 | 0.0340725 | 0.000156841 |
| $bcd \to cad$ | −0.014402 | −0.0389897 | −0.0245877 | $7.47514e-005$ |
| $bcd \to Kr$ | 0.0576209 | 0.0287058 | −0.0289151 | 0.000306426 |
| $bcd \to gt$ | 0.0957429 | 0.0223168 | −0.0734261 | $6.08573e-005$ |
| $bcd \to kni$ | −0.000129675 | 0.0630306 | 0.0631603 | 0.000139936 |

Table 3: Comparison of an average network of the two groups with oscillatory pattern (group III vs. group IV). Interactions that are not significantly different between the two groups are shown in light gray. The interactions that are significantly different are shown in colour. The table summarizes the list of parameters that are significantly different.

# 4 Correlation analysis

One simple approach to explore the parameter determinability is to use cross-correlation between parameters [19]. A correlation matrix shows the degree of association between two parameters. The parameter values are centered on the mean and the normalised cross-correlation between −1 and +1 is computed using the Pearson correlation. From the inverse modelling paradigm, the correlations describe compensation that may arise from an incomplete or inaccurate data set, i.e. the data set does not contain enough information to cover all parameters. Compensation may however also arise from an incomplete model, i.e. the model does not sufficiently represent the underlying biological mechanism. Typically compensation can occur if the time derivative, or gene change rate is remaining the same, while changing different parameters. Examples of these are the promoter rates $R$ and the decay rates $\lambda$, which both scale the expression profile, but in different directions and in general show strong correlation patterns. Furthermore, the input weights on a single gene can also compensate each other. If a positive input on a gene becomes stronger, increasing negative weights or decreasing positive weights can adjust for the increased total input, such that the total input on that gene is not altered much. However, these correlation patterns are quite variable and difficult to predict and strongly depend on the precise spatial pattern. From the correlation matrix obtained from the 101 gap gene circuits shown in Figure. **??**, we see the intricateness of the correlation patterns. Considering only absolute correlation values ($|r| \geq 0.6$), cross-correlation are classified as follow:

1. direct correlations: (involving a gene regulators)
   (a) negative correlations between a gene's activators when their contribution is partially on the same anterior-posterior (A/P) domain.
   (b) negative correlations between a gene's repressors when their contribution is partially on the same (A/P) domain.
   (c) positive correlation between a gene's activators vs. its repressor when their contribution is partially on the same (A/P) domain.
   (d) co-correlation caused by the domain geometry (boundary control mainly). Usually, it is regulatory interactions of two different parameters on a gene having the same function (activation or repression) but acting on non-overlapping (A/P) domain.
2. indirect correlation caused by the profile variation.

**Production rate and decay** Systematically for all genes but *tll*, strong negative correlation is observed between all pairs of production rate and decay coefficients ($\mathrm{r}(R_a/\lambda_a) \geq 0.65$). The strong linear correlation represents the scaling of the expression profile. If one increases the production rate of a gene $a$ and wants to keep the system in its normal expression level, one has to decrease the decay related to the protein half-life of the product of gene $a$. Figure. 2 illustrates the negative pairwise production/decay correlation of the genes *hb* and *kni*.

**Gene regulatory parameters** In classical micro-array data analysis, a direct correlation exists between the regulator-regulatee relationships. This association is described if a set of genes (regulatees) increases or decreases their protein level with the increase or decrease of the expression of another group of genes (regulators). In the current context, we see the same behaviour at the parametric level. It is necessary to discriminate between interactions that are consequence of an over-fitting and parameters that might suggest a real interaction. First, we describe the different regulatory mechanisms obtained from the 101 gap gene circuits presented in the main document, obtained from [1, 2]. Based on the correlation-matrix, we identify the interactions that have a very large cross-correlation with all (or most) other parameters, implying that it is not possible to trust their significance.

**Caudal** regulation consist mainly of:

- auto-repression.

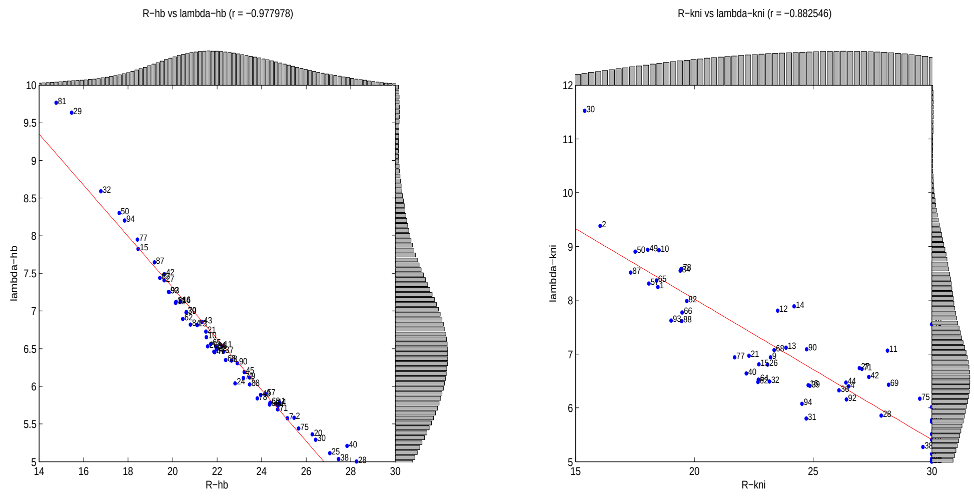


(a) $R_{Hb}/\lambda_{Hb}$ (b) $R_{kni}/\lambda_{kni}$

Figure 2: Scatter plots of production and decay with regression lines and correlation coefficients. At the top and right the estimated parameter distribution is shown, which was calculated using using ksdensity.

- repression by Bcd and Tll.
- negative regulation by Hb, Kr, Gt and kni.

From the dendrogram shown on the right panel of Figure. **??**, we see two main clusters acting on *cad*. The first is composed by $W_{cad}^{cad}$, *cad* promotor threshold $H_{cad}$, production rate $R_{cad}$, and decay $\lambda_{cad}$. In this cluster, a strong negative correlation between *cad* auto-repression and its production rate indicates the compensation effect in order to scale the profile. The second cluster contains *cad* negative regulators (Hb, Gt, Kr and Kni) and maternal influence of Bcd and Cad. In this group, two types of correlations are present:

1. negative correlation between repressors acting on the same domain: ($W_{cad}^{hb}$ vs. $bcd_{cad}$),
2. positive co-correlation of $W_{cad}^{hb}$ vs. $W_{cad}^{kni}$, $W_{cad}^{hb}$ vs. $W_{cad}^{Kr}$ and $W_{cad}^{Kr}$ vs. $W_{cad}^{gt}$). These correlations express geometry maintenance by symmetric action on cad to keep the gene expression level proportional in all domains.

Plots in Figure. 8a-d illustrate the strong correlation of parameters acting on cad.

**hunchback** regulation obtained from the reverse engineering is mainly controlled by the following:

- activation by Bcd and Cad, confirming that they are both the primary activators of the gap domain, acting respectively on the anterior and the posterior.
- auto-repression.
- repression by Kr (weak), Tll (weak), Gt and Kni (strong)
- activation by Kr (weak) , gt and Tll (weak)

The typical correlations shown in Figure. 5 of the parameters regulating Hb are:

1. negative correlation between opposite regulators ($W_{hb}^{bcd}$ vs. $W_{hb}^{kni}$) and (positive $W_{hb}^{gt}$ vs. $W_{hb}^{kni}$)

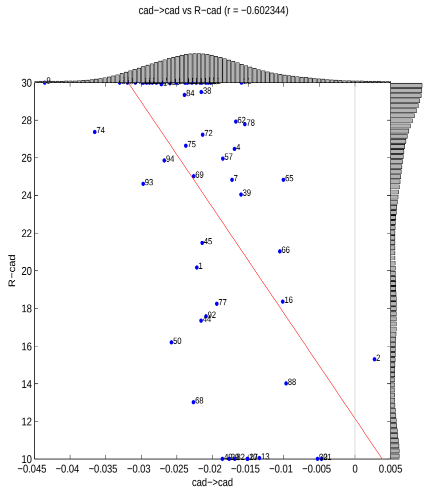


(a) $W_{cad}^{cad}$ / $R_{cad}$

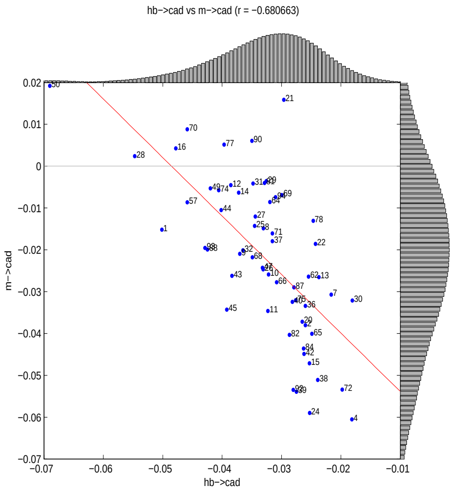


(b) $W_{cad}^{hb}$ / $bcd_{cad}$

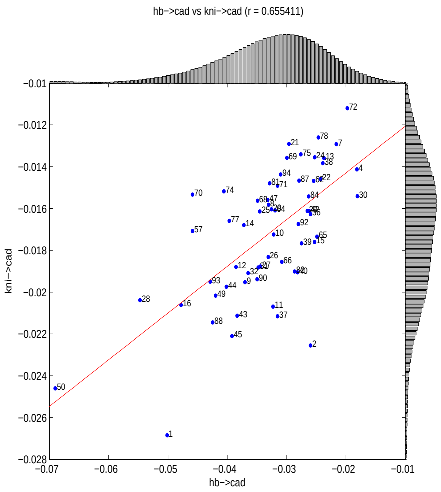


(c) $W_{cad}^{hb}$ / $W_{cad}^{kni}$

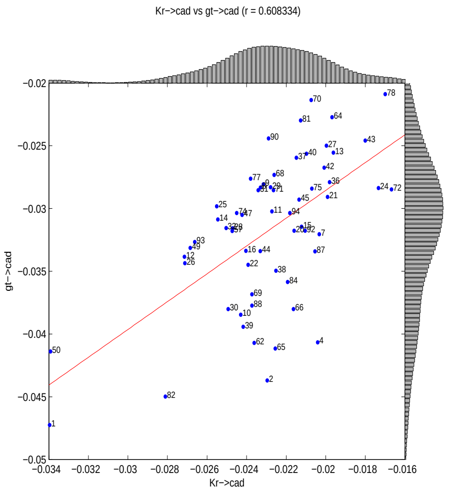


(d) $W_{cad}^{Kr}$ / $W_{cad}^{gt}$

Figure 3: Scatter plots of parameters that regulate Cad gene expression. Only the scatter plots are shown with pairwise correlations higher than 0.6.

2. positive correlation between regulators with opposite functionality on the same domain on a gene ( $W_{hb}^{gt}$ vs. $bcd_{hb}$ and $W_{hb}^{Kr}$ vs. $bcd_{hb}$)

3. co-correlation caused by the domain geometry ($W_{hb}^{cad}$ vs. $W_{hb}^{hb}$)

Perkins et al. [20] suggested that the posterior of Hb is activated by Tll while Jaeger et al. [1] found that posterior Hb is activated by Cad. We found that Gt and Tll have both positive and negative regulatory parameters on *hb*. Assuming that posterior Hb is also activated by Tll, we were expecting to see negative correlation between Cad and Tll regulation on *hb*. Surprisingly, it was not the case, and *hb* regulation by Tll did not show any particular correlation with any other parameter. In fact, it shows very weak correlation with most of the other parameters implying that this parameter is well determined.

**krüppel** . From the parameter estimates, the different regulatory mechanisms that control Kr gene expression dynamic is defined by:

- maternal activation by Bcd and Cad.
- auto activation.
- repression by Hb, Gt, Kni and Tll.
- activation by Hb and kni.

The correlations shown in Figure. 5 with a meaningful value are the following:

1. negative correlations between kr's repressors when their contribution is mostly on overlapping domain : $W_{Kr}^{hb}$ vs. $W_{Kr}^{gt}$ at the anterior domain and $W_{Kr}^{gt}$ vs. $W_{Kr}^{kni}$ at the posterior domain.

2. negative correlation between $W_{Kr}^{kr}$ vs. $W_{Kr}^{hb}$ and $W_{kr}^{kni}$ (decrease repression weight if auto-activation is weaker)

3. positive correlation between activators vs. repressor when their contribution is mostly on the same domain : $W_{Kr}^{hb}$ vs. $R_{Kr}$ , $W_{Kr}^{kni}$ vs. $R_{Kr}$, $W_{Kr}^{Kr}$ vs. $W_{Kr}^{gt}$ and $W_{Kr}^{hb}$ vs. $W_{Kr}^{kni}$ (*NB: kr auto-activation and production contribute in the entire domain*).

4. positive co-correlation caused by the domain geometry: $W_{kr}^{hb}$ vs. $W_{kr}^{kni}$

Jaeger et al. [1] suggested stronger influence of Bcd than Cad and found $bcd_{kr} \geq W_{kr}^{cad}$. We find equivalent weight for $W_{kr}^{cad}$ and $bcd_{kr}$. However, we did not estimate the total contribution of the gene's parameter and the gene's product. It is suggested that Hb activates anterior kr. The resulting gap gene circuits found both role activation (very weak) and repression. The strong correlation between $W_{kr}^{hb}$ and $W_{kr}^{kni}$ suggests that if one repression increases, the other one also has to increases in order to maintain symmetry and to avoid domain expansion on one side. This result confirms Jaeger et al. [1] hypothesis suggesting that Hb and Kni contribute in the establishment of *kr* border. Hb represses the anterior border while kni represses the posterior border.

**giant** From the 101 circuits obtained, mechanism controlling *gt* is as follow:

- maternal activation by Bcd and Cad.
- auto-activation.
- repression by Hb, Kr, kni and Tll.
- activation by Hb and kni ( very weak).

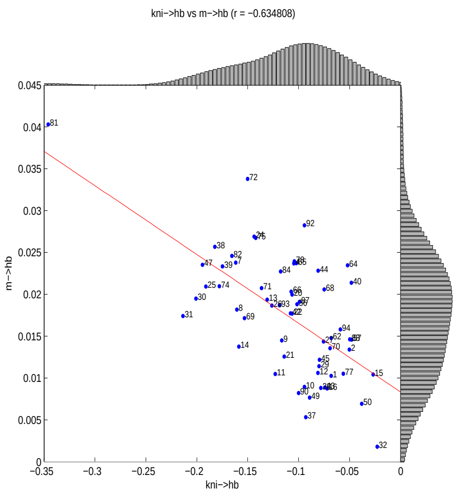


(a) $W_{hb}^{kni}$ / $bcd_{hb}$

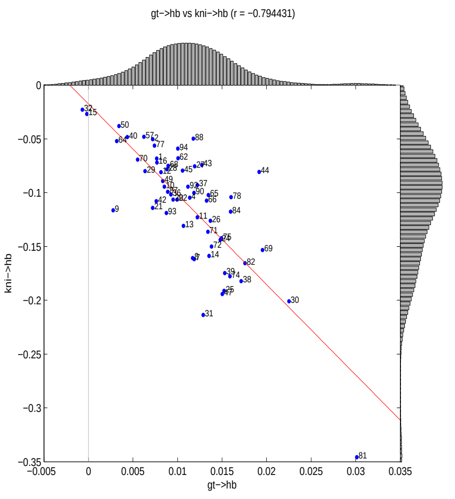


(b) $W_{hb}^{gt}$ / $W_{hb}^{kni}$

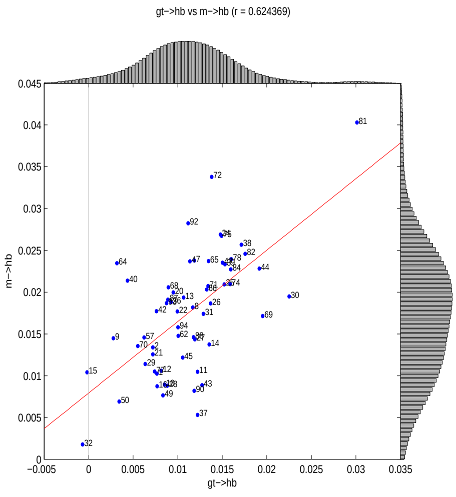


(c) $W_{hb}^{gt}$ / $bcd_{hb}$

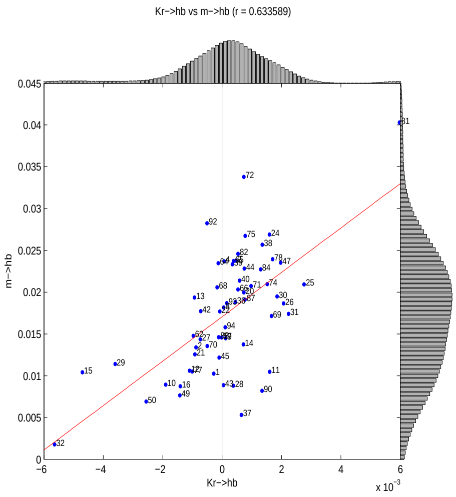


(d) $W_{hb}^{kr}$ / $bcd_{hb}$

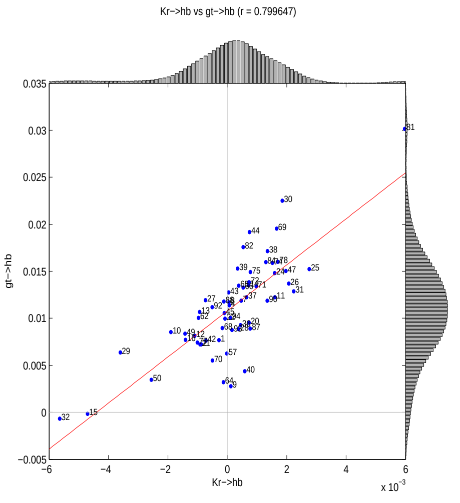


(e) $W_{hb}^{kr}$ / $W_{hb}^{gt}$

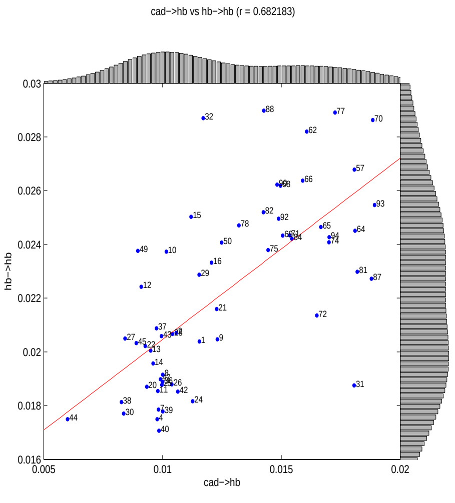


(f) $W_{hb}^{cad}$ / $W_{hb}^{hb}$

Figure 4: Scatter plots of parameters that regulate Hb

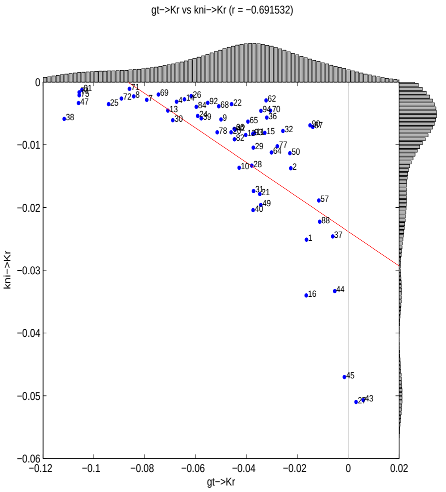


(a) $W_{Kr}^{gt}$ / $W_{Kr}^{kni}$

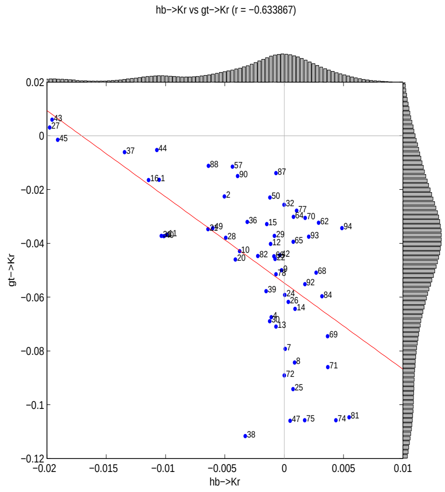


(b) $W_{Kr}^{hb}$ / $W_{Kr}^{gt}$

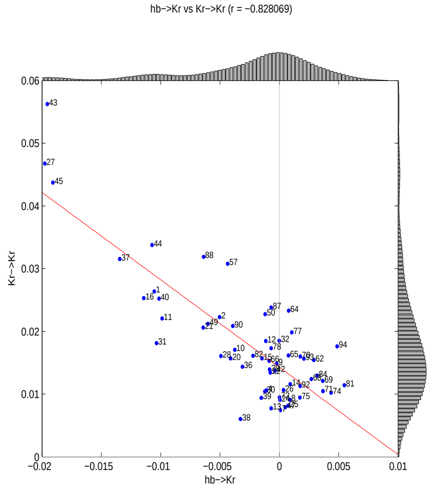


(c) $W_{Kr}^{hb}$ / $W_{Kr}^{Kr}$

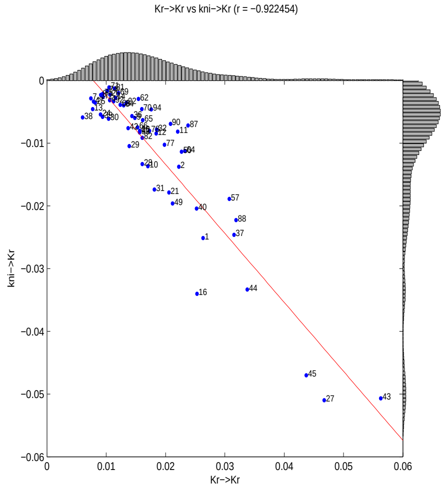


(d) $Kr_{Kr}^{Kr}$ / $W_{Kr}^{kni}$

Figure 5: Scatter plots of parameters that regulate Kr.

Bcd and Cad contribute respectively in the expression of anterior and posterior *gt*. Only two significant correlations (shown in Figure. 6) were found: negative correlations between $W_{gt}^{Kr}$ and $bcd_{gt}$ and between $W_{gt}^{hb}$ and $bcd_{gt}$. The central domain of *gt* regulation is mainly repressed by Kr and the negative correlation translates the balance between decreasing repression and decreasing activation. Although Hb role on *gt* seems weak ( $|W_{gt}^{hb}| \leq 0.005$), the correlation with $bcd_{gt}$ shows that Hb represses anterior giant as suggested by Jaeger et al. [1]. When Hb positively regulates *gt*, Hb mainly contributes in the expression of posterior *gt*. This is observation is confirmed by the negative correlation between repression of gt by Tll and the regulation of gt by Hb for the case where $W_{gt}^{hb} \geq 0$.

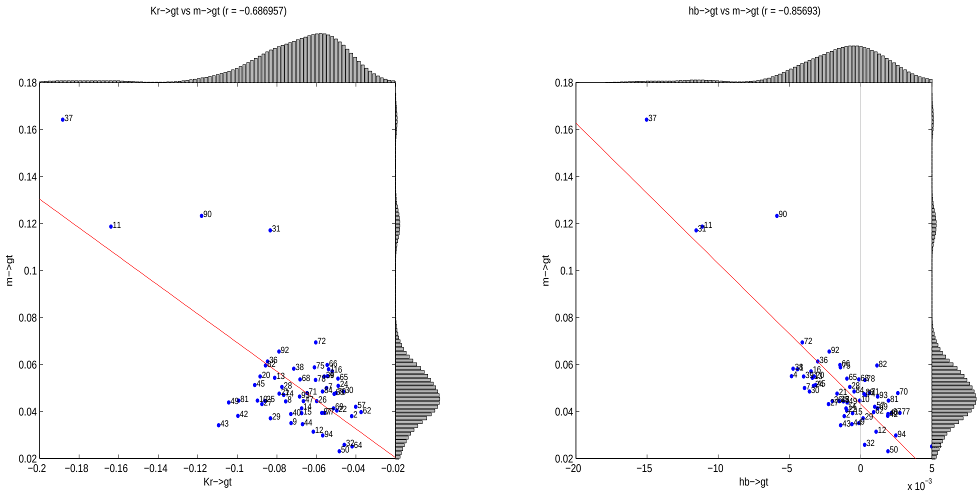


(a) $W_{gt}^{kr}$ $/bcd_{gt}$ (b) $W_{gt}^{hb}$ $/bcd_{gt}$

Figure 6: Scatter plots of parameters that regulate gt.

**knirps** regulation obtained from the gap gene circuits is described as follow:

- maternal activation by Cad and bcd.
- maternal repression by Bcd.
- auto-activation.
- activation by kr and gt
- repression by Hb, Kr, gt and Tll.

The main correlations with a significant Pearson value are the following:

1. negative correlations between $W_{kni}^{cad}$ vs.$W_{kni}^{gt}$, and $W_{kni}^{gt}$ vs. positive $W_{kni}^{kni}$.
2. positive correlation between $W_{kni}^{hb}$ vs. $W_{kni}^{kni}$, $W_{kni}^{gt}$ vs. $R_{kni}$ and $W_{kni}^{Kr}$ vs. $R_{kni}$ .
3. co-correlation caused by the domain geometry between $W_{kni}^{Kr}$ vs. $W_{kni}^{gt}$.

1 and 2 are direct correlations related to compensation phenomena to maintain the expression level. Jaeger et al. [1] proposed that *kni* anterior border is set by repression by Hb and Kr, and posterior border is controlled by Gt and Tll. They also pointed that Kr might not be necessary in the regulation of kni. We found that in 100% of gap gene circuits, *kni* is repressed by Hb and Tll, but it is not systematically repressed by Gt and Kr. $W_{kni}^{Gt}$ and$W_{kni}^{Kr}$ have a similar distribution and seems to have the same role on kni. The very strong positive correlation between $W_{kni}^{kr}$ and $W_{kni}^{gt}$ confirms this

hypothesis and indicates the role of both parameter in maintaining domain symmetry of *kni* to avoid domain expansion.

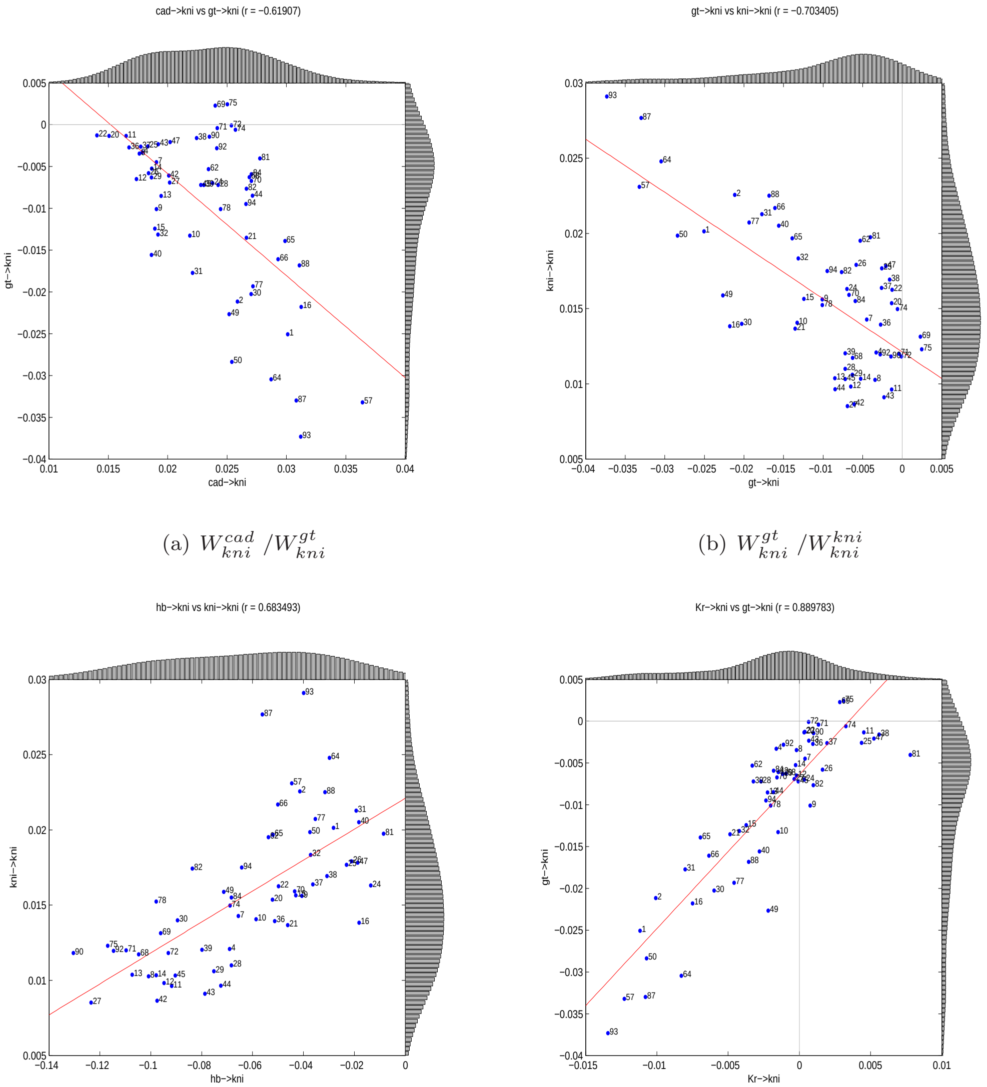


(a) $W_{kni}^{cad}$ $/W_{kni}^{gt}$

(b) $W_{kni}^{gt}$ $/W_{kni}^{kni}$

(c) $W_{kni}^{hb}$ $/W_{kni}^{kni}$

(d) $w_{kni}^{kr}$ $/W_{kni}^{gt}$

Figure 7: Scatter plots of parameters that regulate kni.

**Tailless** regulation obtained from the gap gene circuits is as follow:

- maternal activation by Cad and Bcd.
- maternal repression by cad and Bcd.
- auto-activation.
- activation by Hb, kr, Gt
- repression by Hb, Kr, Gt kni.

The disparities obtained for Tll regulators show that the missing data and probably the missing gene (*hucklebin*) lead to different set of parameter in the search space, making it difficult to interpret. As expected, Cad maternally regulates Tll. Cad weights are weak and this is explained by the high level of Cad on the posterior domain of the embryo. The total transcription factor of Cad on Tll has a very large contribution. Contrarily to the gap gene, in most cases Bcd represses Tll.

**Diffusion** In [1], Jaeger et al. it was shown that diffusion does not consistently contributes in the expression of the shift domain. We did not find systematic strong correlation between diffusion and any other parameters beside Kr and Gt. Their auto-activation parameters are respectively positively correlated to the diffusion coefficient ($r(W_{gt}^{gt}/D_{gt})$ = 0.605 and $r(W_{Kr}^{Kr}/D_{Kr})$ = 0.64). If their gene concentration is increased by means of auto-regulation, the amount of protein diffusing should also increase. Although these correlations are obvious, we cannot explain why a similar feature is not present for *hb, kni*, and *tll*. In contrary, the others diffusion parameters have very weak correlation with any other parameters, signifying that the diffusion coefficient can be determined from the current model with the available data.

**Geometry based co-correlations** Clustering the parameters reveals a group composed of Cad activation on *hb, kr, gt* and *kni*. All these parameters are strongly positively correlated ($W_{hb}^{cad}$ vs. $W_{kni}^{cad}$, $W_{gt}^{cad}$ vs. $W_{kni}^{cad}$, $W_{hb}^{cad}$ vs. $W_{Kr}^{cad}$, $W_{Kr}^{cad}$ vs. $W_{gt}^{cad}$, $W_{hb}^{cad}$ vs. $W_{gt}^{cad}$ and $W_{Kr}^{cad}$ vs. $W_{kni}^{cad}$ ). These correlations express the maintenance of gap gene profile proportional to each other on the action of Cad.

**Indirect correlations.** Few indirect correlation of type $W_b^a$ vs. $W_d^c$ mainly caused by profile variation are present. These correlations are mainly related to Tll regulation such as: $W_{kni}^{Tll}$ vs. $bcd_{Tll}$, $WTll_{Kr}$ vs. $bcd_{Tll}$, $W_{Kr}^{Tll}$ vs. $W_{kni}^{Tll}$, $W_{Kr}^{Tll}$ vs. $W_{kni}^{cad}$ and $W_{Kr}^{Tll}$ vs. $W_{Tll}^{gt}$. Also there is a positive correlation between $bcd_{Kr}$ and $bcd_{gt}$. This indirect correlation is caused by the mutual repression between of Kr and gt. The change in the repressive parameter is balanced by the Bcd. If one repressor increase/decrease, the mutual repressor acts in an similar manner. Consequently, the maternal influence is adjusted to keep the gene expression at the desire level.

**Influence of the promoter threshold** We also observe a strong negative correlation between $W_{cad}^{cad}$ and its promotor threshold suggesting that the level of auto-activation or auto-repression is clearly linked to the threshold. Another interesting type of correlations is the one between Tll promoter threshold with some of the regulators ($W_{Tll}^{hb}$ vs. $h_{Tll}$ and $W_{Tll}^{cad}$ vs. $h_{Tll}$). Therefore, one cannot conclude that it is a strong or weak action just by focusing on the weight of the parameter given that the level of production depends on the threshold [21].

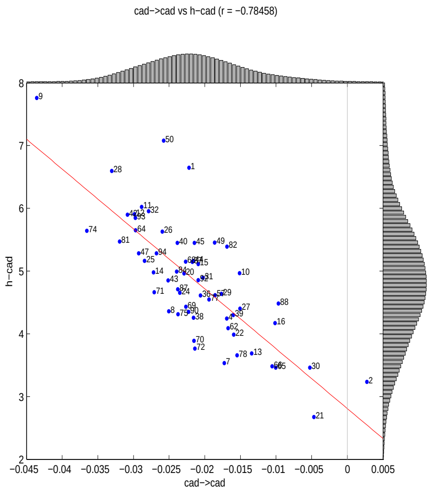


(a) $W_{cad}^{cad} \ /h_{cad}$

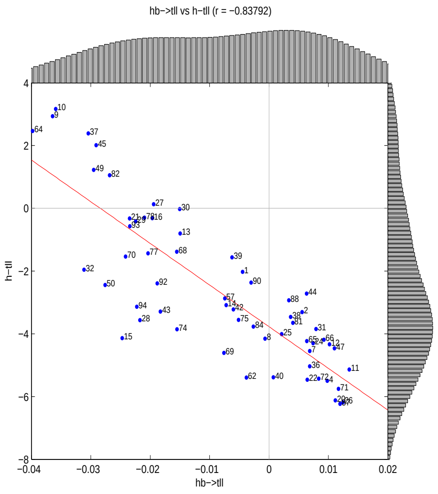


(b) $W_{Tll}^{hb} \ /h_{Tll}$

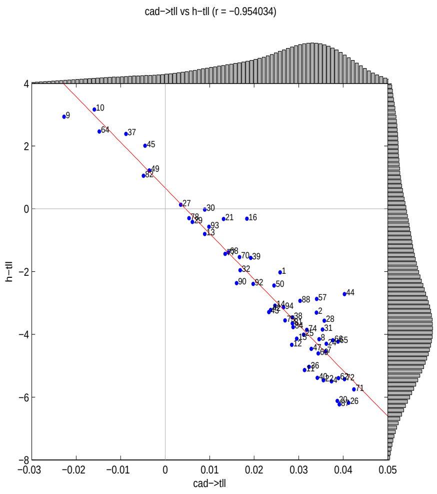


(c) $W_{Tll}^{cad} \ /h_{Tll}$

Figure 8: Scatter plots of parameters that regulate caudal. Only the scatter plots with pairwise correlations higher than 0.6 are shown.

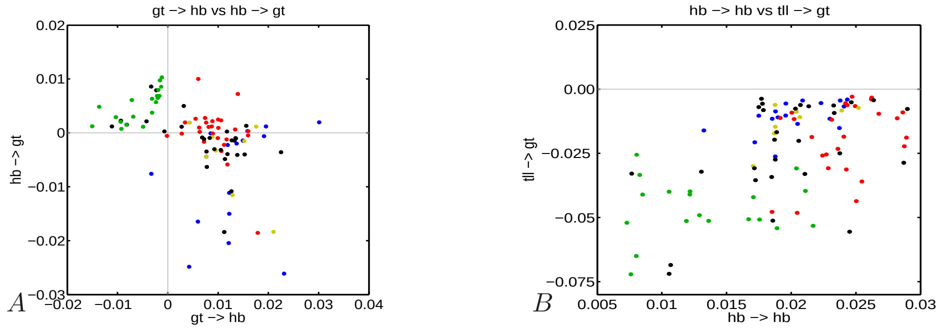


Figure 9: Scatter plots of different parameters that are significantly different between different stability groups. The colors indicate the different stability groups: fixed pattern in yellow (Group I), fixed pattern with large *hb* domain in red (Group II), oscillatory group in blue (Group III), oscillatory group in green (Group IV) and other types that could not be classified in the above groups in black.

# References


[1] Jaeger J, Surkova S, Blagov M, Janssens H, Kosman D, Kozlov KN, Myasnikova E, Vanario-Alonso CE, Samsonova M, Sharp DH, Reinitz J: **Dynamic control of positional information in the early Drosophila embryo.** *Nature* 2004, **430**(6997):368 – 371.

[2] Fomekong-Nanfack Y, Kaandorp JA, Blom J: **Efficient parameter estimation for spatio-temporal models of pattern formation: case study of Drosophila melanogaster.** *Bioinformatics* 2007, **23**(24):3356–3363.

[3] Kosman D, Small S, Reinitz J: **Rapid preparation of a panel of polyclonal antibodies to Drosophila segmentation proteins**. *Dev. Genes Evol.* 1998, **208**:290–294.

[4] Myasnikova E, Kosman D, Reinitz J, Samsonova M: **Spatio-Temporal Registration of the Expression Patterns of Drosophila Segmentation Genes**. In *Proceedings of the Seventh International Conference on Intelligent Systems for Molecular Biology*, AAAI Press 1999:195–201.

[5] Myasnikova EM, Samsonova AA, Samsonova MG, Reinitz J: **Spatial registration of in situ gene expression data**. *Molecular Biology* 2001, **35**(6):955–960.

[6] Myasnikova E, Samsonova A, Kozlov K, Samsonova M, Reinitz J: **Registration of the expression patterns of Drosophila segmentation genes by two independent methods.** *Bioinformatics* 2001, **17**:3 – 12.

[7] Foe VE, Alberts BM: **Studies of nuclear and cytoplasmic behaviour during the five mitotic cycles that precede gastrulation in Drosophila embryogenesis.** *J. Cell. Sci.* 1983, **61**:31 – 70.

[8] Chu KW, Deng Y, Reinitz J: **Parallel simulated annealing by mixing of states**. *J. Comput. Phys.* 1999, **148**(2):646–662.

[9] Lam J, Delosme JM: **An Efficient Simulated Annealing Schedule: Derivation**. Tech. Rep. 8816, Electrical Engineering Department, Yale, New Haven, CT 1988.

[10] Lam J, Delosme JM: **An Efficient Simulated Annealing Schedule: Implementation and Evaluation**. Tech. Rep. 8817, Electrical Engineering Department, New Haven, CT 1988.

[11] Runarsson TP, Yao X: **Stochastic Ranking for Constrained Evolutionary Optimization**. *IEEE Transactions on Evolutionary Computation* 2000, **4**(3):284–294.

[12] Runarsson TP, Yao X: **Search biases in constrained evolutionary optimization**. *IEEE Transactions on Systems, Man, and Cybernetics, Part C* 2005, **35**(2):233–243.

[13] Hooke R, Jeeves TA: **Direct search solution of numerical and statistical problems**. *J. Assoc. Comput. Mach.* 1961, **8**:212–229.

[14] Nelder J, Mead R: **A simplex method for function minimization**. *Computer Journal* 1965, **7**:308 313.

[15] Lewis RM, Shepherd A, Torczon V: **Implementing generating set search methods for linearly constrained minimization**. Tech. Rep. WM–CS–2005–01, Department of Computer Science, College of William & Mary 2005. [Revised July 2006].

[16] Reinitz J, Sharp DH: **Mechanism of eve stripe formation.** *Mech. Dev.* 1995, **49**(1-2):133 – 158.

[17] D'haeseleer P: **How does gene expression clustering work?** *Nature Biotechnology* 2005, **23**:1499 – 1501.

[18] Eisen MB, Spellman PT, Brown PO, Botstein D: **Cluster analysis and display of genome-wide expression patterns.** *Proc Natl Acad Sci U S A* 1998 Dec 8, **95**(25):14863–14868.

[19] Jaqaman K, Danuser G: **Linking data to models: data regression.** *Nat Rev Mol Cell Biol* 2006 Nov, **7**(11):813–819.

[20] Perkins TJ, Jaeger J, Reinitz J, Glass L: **Reverse engineering the gap gene network of Drosophila melanogaster.** *PLoS Comput. Biol.* 2006, **2**(5):e51.

[21] Ashyraliyev M, Jaeger J, Blom JG: **Parameter estimation and determinability analysis applied to Drosophila gap gene circuits**. *BMC Systems Biology* 2008, **2**(83).
